# Validation of Methods to Control for Immortal Time Bias in a Pharmacoepidemiologic Analysis of Renin–Angiotensin System Inhibitors in Type 2 Diabetes

**DOI:** 10.2188/jea.JE20130164

**Published:** 2014-07-05

**Authors:** Xilin Yang, Alice PS Kong, Andrea OY Luk, Risa Ozaki, Gary TC Ko, Ronald CW Ma, Juliana CN Chan, Wing Yee So

**Affiliations:** 1 Department of Medicine and Therapeutics, The Chinese University of Hong Kong, Prince of Wales Hospital, Hong Kong SAR, China; 2 Department of Epidemiology and Biostatistics, School of Public Health, Tianjin Medical University, Tianjin, China; 3 Li Ka Shing Institute of Health Sciences, The Chinese University of Hong Kong, Prince of Wales Hospital, Hong Kong SAR, China; 4 Hong Kong Institute of Diabetes and Obesity, The Chinese University of Hong Kong, Prince of Wales Hospital, Hong Kong SAR, China

**Keywords:** cardiovascular disease, immortal time bias, renin–angiotensin system inhibitors, time-dependent Cox model, cancer, type 2 diabetes

## Abstract

**Background:**

Pharmacoepidemiologic analysis can confirm whether drug efficacy in a randomized controlled trial (RCT) translates to effectiveness in real settings. We examined methods used to control for immortal time bias in an analysis of renin–angiotensin system (RAS) inhibitors as the reference cardioprotective drug.

**Methods:**

We analyzed data from 3928 patients with type 2 diabetes who were recruited into the Hong Kong Diabetes Registry between 1996 and 2005 and followed up to July 30, 2005. Different Cox models were used to obtain hazard ratios (HRs) for cardiovascular disease (CVD) associated with RAS inhibitors. These HRs were then compared to the HR of 0.92 reported in a recent meta-analysis of RCTs.

**Results:**

During a median follow-up period of 5.45 years, 7.23% (*n* = 284) patients developed CVD and 38.7% (*n* = 1519) were started on RAS inhibitors, with 39.1% of immortal time among the users. In multivariable analysis, time-dependent drug-exposure Cox models and Cox models that moved immortal time from users to nonusers both severely inflated the HR, and time-fixed models that included immortal time deflated the HR. Use of time-fixed Cox models that excluded immortal time resulted in a HR of only 0.89 (95% CI, 0.68–1.17) for CVD associated with RAS inhibitors, which is closer to the values reported in RCTs.

**Conclusions:**

In pharmacoepidemiologic analysis, time-dependent drug exposure models and models that move immortal time from users to nonusers may introduce substantial bias in investigations of the effects of RAS inhibitors on CVD in type 2 diabetes.

## INTRODUCTION

With the availability of electronic medical records, many researchers are using large drug databases to perform pharmacoepidemiologic analyses.^1^ However, the results of these analyses often result in more controversy than clarity.^2^ A major challenge in analyzing data collected in observational cohorts is the random and often incomplete nature of collection of information on confounders, as well as bias from various sources. Although randomized controlled trials (RCTs) are often considered the gold standard in evaluating drug efficacy, there are many challenges in such studies, including cost implications in conducting large-scale comparative trials in populations with heterogeneous clinical profiles and outcomes. Thus, appropriate analyses of databases containing quality data collected in real-world settings could be useful in validating findings from efficacy trials in a less-controlled setting, guiding clinical practice, identifying unmet needs, and generating hypotheses for further testing.

In cohort studies, drug therapies may be started at any time during follow-up. If a drug is started after study enrollment, the period from enrollment to the start of drug therapy is considered “immortal” since the patient must be alive before the drug is started. Similarly, if a patient develops the study endpoint before the start of drug therapy, he/she will be considered a nonuser even though he/she is subsequently treated with the drug. Immortal time bias refers to bias due to misclassifying the non-exposure period before drug commencement as the exposure period during analysis.

A common approach to control for immortal time bias is to remove it in drug users. However, some drug users may have had the outcome during the immortal time period. Because use of the drug after the outcome could affect the occurrence of the outcome, this group of drug users is classified as drug nonusers. Suissa^3^ maintained that exclusion of immortal time from users may lead to overestimation or inflation of the rate of the outcome among nonusers and that immortal time should be added to the denominator of the nonusers, without addition of any new events of the outcome to the numerator, to avoid a downward-biased risk ratio of the drug for the outcome.

Another widely advocated method to control for immortal time bias is to use time-dependent analysis of drug exposure during follow-up.^3^^–^^5^ This method requires that the drug be prescribed at random,^6^ but this rarely occurs in practice since there are always drug indications that may not be captured in the dataset. Type 2 diabetes (T2D) is characterized by gradual deterioration of metabolic control—including hyperglycemia and development of other risk conditions such as hypertension, albuminuria, dyslipidemia, and renal dysfunction^7^—resulting in increased drug use among people with the disease. Thus, the association of drug exposure with T2D outcomes is heavily confounded by the close associations among cardiometabolic risk factors (often not measured). In the absence of RCT data, methods used to control bias during pharmacoepidemiologic analysis should be validated, since a flawed methodology could lead to erroneous conclusions and negative effects on research direction and clinical practice. In accordance with this proposition, we systematically applied these proposed methods to estimate the hazard ratio (HR) for cardiovascular disease (CVD) associated with renin–angiotensin system (RAS) inhibitors. We sought to determine whether the HRs in these models would fall within the 95% confidence interval (CI) of 0.84 to 1.00 for the HR of 0.92 for CVD associated with RAS inhibitors, which was reported in a recent meta-analysis of RCTs of the effect of RAS inhibitors on CVD in patients with T2D.^8^

## METHODS

### The patients

The study cohort and methodology, including laboratory assays, have been previously described.^9^^,^^10^ Briefly, data from the Hong Kong Diabetes Registry were used in this analysis. The Registry was established in 1995 at the Prince of Wales Hospital as part of a quality improvement program and covers a population of over 1.2 million. The Hong Kong Government maintains a heavily subsidized healthcare system, and most patients with chronic illnesses, including diabetes, are managed in hospitals governed by the Hospital Authority, which provides more than 95% of acute and chronic care.^10^ People with Type 2 diabetes in the study cohort were referred from general practitioners, community clinics, and specialty clinics of the Hospital Authority and included patients discharged from the Prince of Wales Hospital and other hospitals. As of December 2007, 10 129 patients were enrolled in the Registry. We selected 7387 patients who were enrolled during the period from December 1996 to January 2005 and had detailed data on drugs commonly used to treat diabetes. We sequentially excluded 328 patients with type 1 diabetes (including patients with missing data on diabetes type), 45 of non-Chinese ethnicity, 1135 with a history of CVD before enrollment, and 608 with missing data for other variables. We further excluded 1343 patients who had been exposed to RAS inhibitors during the 2.5 years before enrollment, as drug exposure 2.5 years before enrollment had little impact on the estimated HR for clinical outcomes associated with drug use.^11^ Thus, 3928 patients were included in the current analysis. The definition of CVD at enrollment was previously described.^12^

At baseline, and regularly thereafter, the enrolled patients with T2D underwent a comprehensive 4-hour assessment of diabetes-related complications and risk factors, which was based on the European DiabCare protocol.^13^ The assessment included an interview by diabetes nurses, anthropometric measurements, biochemical evaluation, fundus examination, and podiatry assessment. After an overnight fast of at least 8 hours, blood samples were collected from patients to assay fasting lipids, glucose, and HbA_1c_ and for renal and liver function tests. A random spot urine sample was collected to measure albumin-to-creatinine ratio (ACR). Albuminuria was defined as a urinary ACR of 2.5 mg/mmol or higher in men or 3.5 mg/mmol or higher in women. The abbreviated Modification of Diet in Renal Disease Study (MDRD) formula, recalibrated for Chinese,^14^ was used to calculate estimated glomerular filtration rate (eGFR). Informed written consent was obtained from all participants, and the study was approved by the Chinese University of Hong Kong Clinical Research Ethics Committee.

### Definition of CVD

Incident CVD events were defined by the principal discharge diagnosis or principal procedure codes, including myocardial infarction, ischemic heart disease, coronary revascularization, percutaneous transluminal coronary angioplasty, coronary atherectomy, stroke, and peripheral arterial disease, based on the International Classification of Diseases, Ninth Revision.^12^

### Statistical analysis

The Statistical Analysis System (Release 9.30) was used to perform the statistical analysis (SAS Institute Inc., Cary, NC, USA), unless specified. All data are expressed as median (interquartile range; IQR) or mean ± SD, as appropriate, and were analyzed accordingly. Follow-up time was calculated as the period in years from enrollment to the date of the first CVD event, death, or censoring date, ie, July 30, 2005, whichever came first. Cox proportional hazards regression was used to estimate the HR and 95% CI for CVD associated with the use of RAS inhibitors.

We used an HR of 0.92 (95% CI, 0.84–1.00) as the “gold standard,” as it had been reported in a meta-analysis of RCTs of the cardioprotective effects of RAS inhibitors.^8^ To identify potential bias related to immortal time, we used a time-fixed Cox model with inclusion of immortal time as the first method to estimate the HR. Because Suissa^3^ recommended using a time-dependent Cox model to control for immortal time, we used this model as our second validation method. In the analysis, we coded use of RAS inhibitors as 0 for nonuse of the drug before their use and as 1 for use since the start of RAS inhibitor treatment. On the basis of the suggestion of Suissa,^3^ the third method for validation was a Cox model with exclusion of immortal time for users and inclusion of it for nonusers. Time 0 of the follow-up among users was assigned to the time RAS inhibitors were started; time 0 among the nonusers was the time of enrollment. Additional analysis used Cox models that excluded immortal time from users and did not add it to nonusers.

RAS inhibitor use during follow-up was defined as use of either angiotensin-converting enzyme inhibitors (ACEIs) or angiotensin II receptor blockers (ARBs). Immortal time was defined as the period in years from enrollment to the date RAS inhibitor treatment was started. A structured scheme was used to adjust for confounders: (1) covariables at enrollment, (2) use of other drugs during follow-up, and (3) re-estimated covariables when RAS inhibitors were started during follow-up (see below). Covariables used in the adjustment included age, sex, use of tobacco and alcohol, occupation, body mass index (BMI), duration of diabetes, HbA_1c_, systolic blood pressure (SBP), low-density lipoprotein cholesterol (LDL-C), high-density lipoprotein cholesterol (HDL-C), triglyceride, natural log-transformed spot urinary albumin: creatinine ratio (ACR+1), eGFR, and use of statins, insulin, metformin, gliclazide, glibenclamide, and thiazolidinediones during follow-up.

Covariables at the time RAS inhibitors were started (or end of immortal time) were estimated from covariables measured at enrollment (See eTable). We applied the intention-to-treat principle for analysis of randomized controlled trial data and ignored whether the drug might or might not be discontinued after commencement during follow-up. Adjusted cumulative hazard plots were obtained from Cox model analysis using the Statistical Package for the Social Sciences for Windows (Release 16.0, SPSS Inc, Chicago, IL, USA) to check whether non-inclusion of the immortal time of users in the risk interval of the nonusers increased hazard during the early phase of follow-up and whether inclusion of immortal time in nonusers led to an unbiased hazard during early follow-up.

Plots of LOG [-LOG (Survival function)] versus LOG (follow-up time in years) were used to check the proportional hazards assumption for categorical variables, while the supremum test was used to check the assumption for continuous variables.^15^ A 2-sided *P* value of less than 0.05 was considered to be statistically significant.

## RESULTS

### Patient characteristics

The cohort had a median age of 54 years (IQR, 44–64) and a median duration of diabetes of 5 years (1–10). During a total of 20 174 years of follow-up and a median follow-up period of 5.45 years (3.09–7.22), 7.23% (*n* = 284), or 14.08 patients per 1000 person-years (95% CI, 12.45–15.74), developed CVD. Patients with CVD were older, had a longer duration of diabetes, had worse metabolic profiles at enrollment (with higher HbA_1c_, SBP, LDL-C, and triglyceride and lower HDL-C), and had higher urinary ACR and lower eGFR than did those without incident CVD. Patients with CVD were also more likely to use RAS inhibitors, statins, metformin, and insulin during follow-up.

During follow-up, 38.7% (*n* = 1519) were started on RAS inhibitors; median follow-up time was 1.48 years (IQR, 0.36–3.37) from enrollment to drug commencement. Total immortal time was 3291.9 person-years, which accounted for 39.1% of the 8409 person-years of follow-up among patients treated with RAS inhibitors. During a total of 11 765 person-years of follow-up, CVD incidence in the RAS inhibitor non-user group was 13.17 per 1000 person-years as compared with 15.34 per 1000 person-years in the user group. After exclusion of immortal time, incidence increased to 25.21 per 1000 person-years in the user group. In contrast, after inclusion of immortal time, incidence decreased to 10.29 per 1000 person-years in the nonuser group. As compared with non-users, RAS inhibitor users were older and had longer duration of diabetes, higher BMI, BP, ACR, and HbA1c, and worse renal function. They were also more likely to use other drugs and to develop CVD (Table 1).

**Table 1.** Clinical and biochemical characteristics of a cohort of 3928 patients with type 2 diabetes stratified according to exposure to RAS inhibitors during follow-up

	RAS inhibitor users (*n* = 1519)	RAS inhibitor nonusers (*n* = 2409)	*P*^a^
	
	Median (25th to 75th) or *n* (%)	Median (25th to 75th) or *n* (%)	
**Baseline variables**			
Age, years	57 (47–67)	51 (42–62)	<0.001
Male gender	695 (45.8%)	1091 (45.3%)	0.776
Occupation			<0.001
Full-time	528 (34.8%)	968 (40.2%)	
Housework	442 (29.1%)	681 (28.3%)	
Retired	400 (26.3%)	477 (19.8%)	
Others	149 (9.8%)	283 (11.8%)	
Smoking status			0.387
Ex-smoker	211 (13.9%)	307 (12.7%)	
Current smoker	232 (15.3%)	399 (16.6%)	
Alcohol intake			0.069
Ex-drinker	179 (11.8%)	250 (10.4%)	
Current drinker	101 (6.7%)	202 (8.4%)	
Duration of diabetes, years	6 (2–11)	4 (1–9)	<0.001
Body mass index, kg/m^2^	25.1 (23.0–27.9)	24.1 (22.0–26.6)	<0.001
Systolic BP, mm Hg	138 (127–151)	125 (115–137)	<0.001
Diastolic BP, mm Hg	78 (70–84)	73 (66–80)	<0.001
Glycated hemoglobin, %	7.5 (6.6–8.8)	7.0 (6.1–8.1)	<0.001
Glycated hemoglobin, mmol/mol	58 (49–73)	53 (43–65)	<0.001
LDL-C, mmol/L	3.24 (2.60–3.87)	3.10 (2.50–3.70)	<0.001
HDL-C, mmol/L	1.23 (1.04–1.48)	1.29 (1.08–1.54)	<0.001
Triglyceride, mmol/L	1.39 (0.97–2.04)	1.20 (0.85–1.74)	<0.001
Urinary ACR (mg/mmol)	3.72 (1.18–14.60)	0.95 (0.53–2.01)	<0.001
eGFR, ml min^−1^ 1.73 m^−2^	105.9 (87.2–127.2)	112.8 (96.5–133.3)	<0.001
**Use of drugs and events during follow-up**			
Statins	615 (40.5%)	512 (21.3%)	<0.001
Metformin	1277 (84.1%)	1591 (66.0%)	<0.001
Gliclazide	701 (46.2%)	982 (40.8%)	<0.001
Glibenclamide	492 (32.4%)	654 (26.8%)	<0.001
Thiazolidinediones	140 (9.2%)	96 (4.0%)	<0.001
Insulin	678 (44.6%)	549 (22.8%)	<0.001
CVD	129 (8.5%)	155 (6.4%)	0.015
Death	106 (7.0%)	144 (6.0%)	0.211

Abbreviations: RAS, renin–angiotensin inhibitors; LDL-C, low-density lipoprotein cholesterol; HDL-C, high-density lipoprotein cholesterol; BP, blood pressure; ACR, albumin:creatinine ratio; eGFR, estimated glomerular filtration rate; ACEIs, angiotensin-converting enzyme inhibitors; ARBs, angiotensin II receptor blockers; CVD, cardiovascular disease.

^a^Derived from Wilcoxon 2-sample test, χ^2^ test, or Fisher’s exact test, where appropriate.

### Use of RAS inhibitors and CVD

In the time-fixed Cox model with inclusion of immortal time, use of RAS inhibitors was associated with a nonsignificant increase in the HR (1.16; 95% CI, 0.92–1.47) in univariable analysis, as compared with non-users. Adjustment for covariables at enrollment and use of other medications during follow-up decreased the HR to 0.66 (95% CI, 0.51–0.86; Table 2), ie, the effect size fell below 0.84, which was the lower limit of the 95% CI of the HR from RCTs.

**Table 2.** Hazard ratios for risk of cardiovascular disease (CVD) associated with use of RAS inhibitors during follow-up among patients with type 2 diabetes who did not use RAS inhibitors for at least 2.5 years before enrollment

Use vs nonuse of RAS inhibitors	Hazard ratio	95% CI	*P*
Reported in meta-analysis of RCTs	0.92	0.84–1.00	
Time-fixed Cox model including immortal time			
Univariable analysis	1.16	0.92–1.47	0.213
Adjusted for covariables at enrollment^a^	0.62	0.48–0.80	<0.001
Further adjusted for other drug use^b^	0.66	0.51–0.86	0.002
Time-dependent Cox model including immortal time			
Univariable analysis	2.45	1.35–2.08	<0.001
Adjusted for covariables at enrollment	1.43	1.10–1.88	0.009
Adjusted for covariables at enrollment among non-RAS inhibitor users or time-dependent covariables in users^c^	1.30	0.99–1.70	0.062
Further adjusted for other drug use^b^	1.42	1.08–1.88	0.013
Time-fixed Cox model excluding immortal time among RAS inhibitor users			
Univariable analysis	1.85	1.46–2.34	<0.001
Adjusted for covariables at enrollment	0.96	0.74–1.25	0.757
Adjusted for covariables at enrollment in non-RAS inhibitor users or at the time of use of RAS inhibitors in users^c^	0.86	0.66–1.12	0.251
Further adjusted for other drug use^b^	0.89	0.68–1.17	0.408
Time-fixed Cox model excluding immortal time among users and adding it among nonusers			
Univariable analysis	2.57	2.02–3.28	<0.001
Adjusted for covariables at enrollment	1.36	1.04–1.78	0.027
Adjusted for covariables at enrollment in non-RAS inhibitor users or at the time of use of RAS inhibitors in users^c^	1.22	0.93–1.60	0.151
Further adjusted for other drug use^b^	1.28	0.97–1.68	0.084

Abbreviations: RAS, renin–angiotensin system; RCT, randomized controlled trial.

^a^Covariables at enrollment included age, sex, occupation, duration of diabetes, body mass index, smoking status, alcohol use, low-density lipoprotein cholesterol, high-density lipoprotein cholesterol, triglyceride, systolic blood pressure, HbA_1c_, natural log-transformed spot urinary albumin: creatinine ratio, and estimated glomerular filtration rate.

^b^Drug use included use of insulin, statins, gliclazide, glibenclamide, and thiazolidinediones from enrollment (or initiation of RAS inhibitors in time-fixed models with exclusion of immortal time) to CVD, death, or July 30, 2005, whichever came first.

^c^Covariables at the time of initiation of RAS inhibitors during follow-up among RAS inhibitor users were estimated using partial coefficients of age and duration of diabetes that were obtained from all other covariables at enrollment.

Using the time-dependent Cox model with inclusion of immortal time, the univariable HR was 2.45 (95% CI, 1.35–2.08). Adjustment for covariables at enrollment decreased the HR to 1.43 (1.10–1.88). If we used re-estimated covariables at the time RAS inhibitors were started, the HR decreased to 1.30 (0.99–1.70). Further adjustment for use of other medications changed the HR to 1.42 (1.08–1.88), ie, the estimates from multivariable time-dependent Cox models were above 1.0, the upper limit of the 95% CI.^8^

Use of the time-fixed Cox model that exclude immortal time without re-including it among nonusers resulted in a HR of 1.85 (1.46–2.34), which decreased to 0.96 (0.74–1.25) after adjustment for covariables at enrollment. If we used the re-estimated covariables at the time of RAS inhibitor use, the HR decreased to 0.86 (0.66–1.12). Further adjustment for use of other medications yielded an HR of 0.89 (0.68–1.17), similar to the HR of 0.92 reported in the recent meta-analysis.^8^

Use of the time-fixed Cox model that excluded immortal time from users and re-included it among nonusers increased the HR to 2.57 (2.02–3.28) in univariable analysis, 1.36 (1.04–1.78) after adjustment for covariables at enrollment, 1.22 (0.93–1.60) after further adjustment for covariables at the time of the start of RAS inhibitors, and 1.28 (0.97–1.68) with further consideration of other drugs used during follow-up. All these HRs were above the upper limit of the reported effect size in the recent meta-analysis.

The adjusted cumulative hazard plot did not show an enhanced hazard during early follow-up, after exclusion of immortal time among the user cohort, but it did show an attenuated hazard after re-inclusion of immortal time of users among nonusers (Figure).

**Figure.**
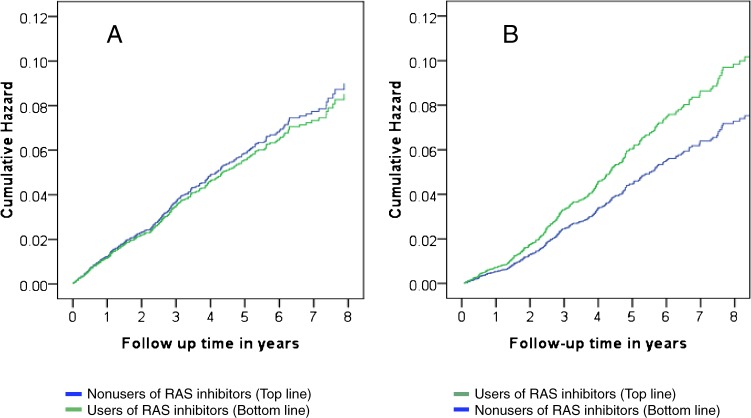
Cumulative hazard of cardiovascular disease (CVD) stratified by use of renin–angiotensin system inhibitors (RAS inhibitors) over time. Legends: A, Exclusion of immortal time among the user cohort without re-inclusion of it among the nonuser cohort; B, Exclusion of immortal time among the user cohort and re-inclusion of it to the nonuser cohort. The 2 plots were adjusted for baseline covariables, shown in Table 2.

## DISCUSSION

In this investigation of a cohort with detailed documentation on CVD risk factors and drug use, we used data on RAS inhibitors, a proven family of cardioprotective drugs, in conjunction with proposed methods to control for immortal time bias to show both the difficulty of removing bias from various sources and the potential to draw erroneous conclusions regarding the effects of RAS inhibitors on CVD. We found that time-dependent drug exposure models and models that moved immortal time from users to nonusers generated HRs far above the upper limit of the 95% CI for the HR for CVD associated with the use of RAS inhibitors from a meta-analysis of RCTs.^8^

The time-dependent Cox model is a common method for removing immortal time bias.^4^^,^^5^ However, our analysis clearly showed that time-dependent Cox models severely inflated the HR for CVD associated with use of RAS inhibitors, leading to the erroneous conclusion that use of RAS inhibitors increased CVD risk. Of course, use of time-dependent models assumes that risk exposure is random.^6^ However, this assumption is severely violated in pharmacoepidemiologic analysis since patients who are started on a drug have a particular risk profile. In the case of RAS inhibitors, the drug indications themselves can influence outcomes, which must be adjusted for accordingly. Even when using a cohort of T2D patients with detailed documentation on baseline risk profiles and re-estimating the risk profile at the time of drug commencement, our adjustment efforts failed to remove major bias due to indications for RAS inhibitor use in the time-dependent drug exposure analysis. In most administrative databases, many major confounders are not available for analysis, which might result in an erroneous conclusion that the drug under study increased the risk of adverse clinical outcomes.

Use of time-fixed Cox models that moved immortal time of drug users to nonusers resulted in HRs that were all above the reference upper limit of the 95% CI of the HR for CVD associated with RAS inhibitors.^8^ Again, adjustment for CVD risk factors attenuated this effect but failed to remove major bias due to the indications for use of RAS inhibitors. Additionally, such a procedure might itself introduce substantial bias, as shown by the clearly attenuated hazard during the early phase of follow-up among nonusers and the artificially inflated HRs for CVD associated with RAS inhibitors. Contrary to the recommendations of Suissa,^3^ excluding immortal time without adding it to the interval at risk for nonusers yielded HRs that all fell within the 95% CI of the HR reported in meta-analysis of RCTs,^8^ without a detectable increase in the hazard of CVD during the early phase among nonusers of RAS inhibitors. Although we cannot entirely exclude the possibility that the correct effect size of RAS inhibitors on CVD from excluding immortal time without re-including it among nonusers might come from balancing the incomplete control of drug use indications with the overestimated risk of CVD among nonusers, the attenuated hazard during early follow-up with models that re-include immortal time among nonusers suggests that CVD risk was underestimated among nonusers, which resulted in inflated HRs for CVD associated with RAS inhibitors.

Confounding by indication^16^^,^^17^ and confounding by immortal time^3^^–^^5^ are major sources of bias in pharmacoepidemiologic analyses. Whether the observed effects of RAS inhibitors on CVD, as determined by different methods, represent true effects depends on how well these approaches simultaneously remove the mixture of confounding effects of drug use indications and immortal time, and on bias from other sources. In addition, a method used to control one source of bias might exaggerate bias from other sources, eg, the time-dependent Cox model worsened confounding by indications for RAS inhibitor use. Although pharmacoepidemiologic analysis is useful for generating new hypotheses in medical research, the results from such analyses must be interpreted with caution, as overreactions to supposedly “novel” findings may have a negative effect on clinical practice.^18^

Our study has several limitations. First, the effect size of use of RAS inhibitors on CVD was based on a meta-analysis of RCTs,^8^ which might not be applicable in real practice. Second, re-estimation of covariables at the time drug therapy began only considered the effects of increasing age and duration of diabetes. We do not know how similar these re-estimated covariables would have been to real levels if they had been measured. Third, this analysis attempted to validate methods for addressing the effects of RAS inhibitors in T2D; applicability to other drugs remains to be confirmed. Last, the present findings were obtained from a single cohort of patients with T2D and need to be confirmed in other similarly designed studies.

In conclusion, we found that use of time-dependent Cox models and time-fixed Cox models with moving of immortal time from drug users to nonusers severely inflated the HR for CVD associated with use of RAS inhibitors. Revealing the caveats of overestimation and underestimation of risks associated with use of different methods in pharmacoepidemiologic analysis highlights the limitations and potentially erroneous conclusions regarding the actual risks of clinical outcomes, including CVD and cancer, associated with use of different drugs in T2D.

## ONLINE ONLY MATERIAL

eTable. Estimated partial regression coefficients of age and duration of diabetes for metabolic indicators at baseline among RAS inhibitor users during follow-up.
