# Supplementary material for: Validation of Methods to Control for Immortal Time Bias in a Pharmacoepidemiologic Analysis of Renin–Angiotensin System Inhibitors in Type 2 Diabetes
Source: J Epidemiol. 2014 Jul 5;24(4):267–73. doi: 10.2188/jea.JE20130164 (PMC4074630; doi:10.2188/jea.JE20130164)
Supplement: eTable. [file je-24-267-s001.pdf]

eTable

We obtained partial regression coefficients for age (β_a) and duration of diabetes (β_b) for demographic and metabolic covariables at enrollment, including HbA_{1c}, BMI, SBP, LDL-C, HDL-C, triglyceride, ACR, and eGFR. We then re-estimated these values at the time when RAS inhibitors were started (X_t), using the following formula: $X_t = X_b + \beta_a T_i + \beta_b T_i$, where X_b is the value at baseline and T_i is the immortal time (See appendix table for β_a and β_b).

eTable. Estimated partial regression coefficients for age and duration of diabetes for metabolic indicators at baseline among RAS inhibitor users during follow-up

**Variables**	**Partial regression coefficient of age**	**Partial regression coefficient of duration of diabetes**
HbA1c,%	-0.00339	0.03902
BMI, kg/m2	-0.06353	0.03979
SBP, mm Hg	0.39584	0.03979
DBP, mm Hg	-0.07609	-0.09017
LDL-C, mmol/L	0.00379	-0.00736
HDL-C, mmol/L	0.00281	-0.00023
Triglyceride, mmol/L	-0.00204	-0.01211
Ln (ACR+1), mg/mmol	-0.00464	0.00544
eGFR, ml min^{-1} 1.73 m^{-2}	-1.32369	-0.22810
